# Risky Bodies in the Plasma Bioeconomy: A Feminist Analysis

**DOI:** 10.1177/1357034X13520331

**Published:** 2015-03

**Authors:** Julie Kent, Anne-Maree Farrell

**Affiliations:** University of the West of England, Bristol, UK; Monash University, Australia

**Keywords:** Anti-D Ig, biopolitics, biotechnology, blood, ethics, pregnancy

## Abstract

In 2003 the UK National Blood Service introduced a policy of ‘male donor preference’ which involved women’s plasma being discarded following blood collection. The policy was based on the view that data relating to the incidence of Transfusion-Related Acute Lung Injury (TRALI) was linked to transfusion with women’s plasma. While appearing to treat female donors as equal to male donors, exclusion criteria operate after donation at the stage of processing blood, thus perpetuating myths of universality even though only certain ‘extractions’ from women are retained for use in transfusion. Many women in the UK receive a plasma-derived product called Anti-D immunoglobulin which is manufactured from pooled male plasma. This article examines ways in which gender has significance for understanding blood relations, and how the blood economy is gendered. In our study of relations between blood donors and recipients, we explore how gendered bodies are produced through the discursive and material practices within blood services. We examine both how donation policies and the manufacturing and use of blood products produces gendered blood relations.

This article examines ways in which gender has significance for understanding blood relations, and how the blood economy is gendered. It has previously been recognised that the wider bioeconomy is gendered through practices which mobilise women’s reproductive tissues, for example, oocytes, fetal tissue, embryos and cord blood for use as sources of stem cells (Kent, 2012; Waldby, 2008; Waldby and Cooper, 2010). Ova exchange for therapeutic treatment of infertility has been framed as enabling women to assist others to have children, but also as reproducing racialised, national ideals (Konrad, 1998; Nahman, 2006). Although data on organ transplantation have identified women as comprising the majority of donors, it also reveals that men are much more likely to become organ recipients (Jindal et al., 2005). What these and other studies have shown is that bodies have become more plastic and flexible, with exchanges of biological materials becoming increasingly associated with new forms of biological citizenship and capital accumulation (Rose and Novas, 2005). In our study of relations between blood donors and recipients we explore how gendered bodies are produced through the discursive and material practices within national blood services.

Anthropological studies of the socio-cultural meanings and ethics of blood donation have drawn attention to the complexities of blood relations and highlighted diversity across communities within differing cultural, national, religious and political contexts (Copeman, 2009; Mauss, 1990 [1950]). Titmuss (1970) claimed that the ethical imperative in whole blood donation through national blood services should be conceptualised using the notion of the gift relationship which involved the altruistic donation of blood without financial reward. This was to be seen as a preferred moral choice underpinned by the social contract that bound communities together. Titmuss’s conception of the gift relationship was underpinned by appeals to universality: it was seen as an important act of social (or biological) citizenship, promoted on the part of national blood services to provide both a moral and institutional basis for encouraging blood donation in an organised manner (Healy, 2006).

A more critical understanding of blood donation and its association with concepts of citizenship, solidarity and altruism has since been developed. Blood donation has been demonstrated to be a highly politicised issue, especially where tensions have arisen due to some social groups being excluded as donors. Policies of ‘donor deferral’, commonly justified by policy makers on the grounds of ensuring blood safety, have been controversial and increasingly subject to critical scrutiny. In practice, the public health objective of ensuring a safe blood supply and underlying ‘safety logics’ (Hoeyer, 2010) has meant that any claim to universality in blood donation is contingent. Donor deferrals and exclusions have been instituted by national blood services for a range of scientific, social and institutional reasons which deserve analysis. Some potential donors are refused permission to donate for reasons that may vary across state boundaries, regulatory regimes or cultural mores. Yet such exclusions are frequently obscured by a discourse of universality of blood donation and under-explored notions of citizenship, as Valentine (2005: 119) points out: ‘The universality of blood is implicitly, and sometimes explicitly, equated with a universal possibility of donating it’, even though exclusions create categories of ‘not quite citizens’. The gendering of bodies has traditionally rendered women as ‘not quite citizens’ or at least different kinds of citizens. While the deferral of men who have sex with men (MSM) from becoming donors has foregrounded links between sexual identity and blood donation (Martucci, 2010; Valentine, 2005), the connections with gender have been less well elaborated. Rather, it is commonly assumed, that (heterosexual) men and women are equally valued as blood donors, especially in western blood economies. We argue here that this is far from the case.

This article focuses primarily on current policy and practices in the UK. It analyses international blood donation policies, clinical and scientific literature, draws on a public tour of a major blood processing facility, a field visit to a plasma products manufacturer and interviews with transfusion specialists, representatives of patient groups and the plasma industry as part of a larger project on consent, risk and safety in UK blood services.^1^ Such research raised questions about contemporary practices within blood services and the plasma industry. Why are all women presumed to represent a threat to the safety of plasma transfusions when not all will become, or will have been, pregnant? How is gender identity mapped onto discourses of risk, safety and understandings of pathological bodies, in this context? Why are men (unless previously transfused), presumed to have low (normal) antibody levels? Given the uncertainties of the causes of a particular complication of plasma transfusion, known as transfusion-related acute lung injury (TRALI), what political conditions contributed to a strategy of excluding women as plasma donors for transfusion in the UK and elsewhere? To what extent have the potential risks of TRALI, associated with transfusion by female plasma, been prioritised over potential benefits, such as the claims that cardiac patients transfused with female plasma had better outcomes?

With these questions in mind, we conclude that blood relations are gendered, transfusion science produces gendered bodies and technologies of plasma fractionation are shaped by gender inequalities. In order to explore this argument, the article first discusses the significance of gender for blood and plasma donation. Then we look at how gender relations structure the production and use of the plasma-derived product Anti-D Immunoglobulin (Anti-D Ig) in the treatment of parturient women to prevent haemolytic disease of the fetus and newborn (HDFN).

## Gender and ‘Risky Bodies’

Feminist analyses have drawn attention to the ways in which gender inequalities are obscured through the universalising narratives of the liberal humanist subject and modernist science. From the 1970s onwards, studies explored aspects of women’s health and elaborated ways in which contemporary biomedicine perpetuated masculinist notions of healthy bodies, neglected the specificities of women’s experience of illness and health, and pathologised pregnancy and childbirth (see, for example, Martin, 1993). Health services have been shown to institutionalise discriminatory practices perpetuating health and social inequalities. Through a focus on embodiment and lived experience, the gendered aspects of health and illness were described. Essentialist readings of the gendered body increasingly became problematised, and understandings of the body as a naturalised, biological given were seen as unsatisfactory. Instead, accounts of the multiple, socio-historical and cultural processes which produce bodies and bodily difference highlighted the contingent and uncertain, leaky boundaries of bodies (Shildrick, 1997). The regulation, disciplining and production of bodies through the biomedical sciences became more clearly recognised as a political project, part of a new kind of ‘vital politics’ or biopolitics (Rose, 2007). Biopower is seen as productive. Following Haraway and others, the entanglement of biology and politics becomes evident, including theories of the immune system (Martin, 1994), transplantation science (Shildrick, 2008), regenerative medicine (Kent, 2012) and, we argue, transfusion science. In short, contemporary theorising of gender raises a number of questions about how gender difference is enacted in blood services through material and discursive practices.

Transfusion science is a discourse or truth regime which creates and translates rules into clinical practices and industry standards in blood services. It underpins not only the organisation and delivery of such services but also the global plasma products industry. Gendered bodies are both naturalised and made invisible in a discourse which draws heavily on universalising myths that seek to value blood and plasma donation as a social and public good, and ties donation to citizenship and social solidarity. It also assumes that bioavailability (Cohen, 2005) has equalising effects whereby all donations are equally valued, despite evidence to the contrary which we will discuss in more detail later.

Our conceptual approach sees policy and practices within blood services and the plasma products industry as situating bodies in different ways – MSM donors, donors with a history of transfusion, donor bodies at risk of variant Creutzfeldt-Jakob Disease (vCJD), female and male donors, and racialised bodies. Blood services and the plasma products industry *produce* difference through the development of categories for sorting, screening, testing, matching and evaluating donor and recipient bodies. White male donor bodies – symbolising the typical blood donor for national blood services – have come to be viewed as superior sources of blood and plasma products.

In the context of meeting increasing demand for blood, scarcity has been an enduring problem for blood services. Attracting a loyal population of repeat donors who give blood on a regular basis is important to address this problem and enhance blood safety (Bonig et al., 2012). There is a significant literature which has examined the motivations and behaviours of donor populations with a view to understanding how best to retain them (Masser et al., 2008). Drawing attention to the numbers of women ineligible to become donors, and evidence that women are less likely to become donors, Healy (2000) sees organisational and cultural factors, rather than individual motivations, as shaping donor populations. The typical blood donor in western blood economies is most commonly male, white and educated, aged in their 30s, with above average occupational level and income (Piliavin and Callero, 1991), and more likely to be involved in civic engagement activities (Alessandrini, 2007).

As discussed previously (Farrell, 2012), techniques for risk assessment and risk management underpin international governance of the blood supply in order to promote blood safety. However, risk is an unstable concept and has been defined in varying ways. Risk assessment has been viewed as the domain of scientific experts and risk management as a matter for political leadership but the role of science and politics in risk governance has been much debated: ‘In simple terms, there are those who view risk as an objective and knowable phenomenon which can be measured, whereas for others it is socially constructed and influenced by cultural, institutional and political contexts’ (Farrell, 2012: 9). We take the view that risk is indeed a socio-cultural construct shaped by political and wider institutional processes. So the use of ‘classificatory technologies’ to identify potential risks to the blood supply must be viewed as one of the ways in which this dynamic operates.

Others have suggested that transplant medicine produces and reifies biocultural categories of racial and ethnic difference through the use of blood group and antigen human leucocyte antigen (HLA) ‘matching criteria’ for organ transplantation in ways that are discriminatory and perpetuate social inequalities (Kierans and Cooper, 2011). We suggest that similar processes are operating within transfusion science and that valuations of bodily materials are variable and gendered. As we will highlight, women’s bodies are constructed as more ‘risky’ in a number of ways by national blood services, such as in relation to the use of female plasma for transfusion and in the manufacture of the blood product, known as Anti-D Ig. In both cases, male bodies are more highly valued in terms of the source material they provide for such blood components and products.

**Figure 1.**
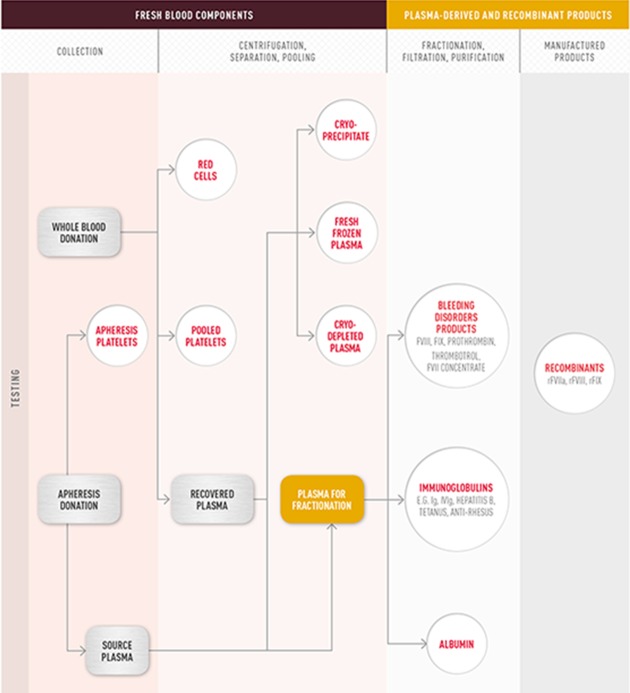
Fresh blood components and plasma-derived and recombinant products. *Source*: www.blood.gov.au/about-blood

The collection and supply of blood for use in the production of a range of components and plasma products has now become a complex undertaking for national blood services (see Figure 1). On the one hand, it is characterised by enduring socio-cultural and political concerns about preferred methods of collection in line with national preferences, which most often crystallises as support for the gift relationship in western bioeconomies. On the other hand, such concerns must be managed in the context of interconnectedness both within and across national boundaries, in relation to collection and supply (Waldby and Mitchell, 2006). This shifts over time and space in the face of emerging risks to blood safety, as well as in relation to changing market demands for blood components and plasma products. Further layers of complexity are added to the mix because collection and manufacturing practices differ between blood components or plasma products, and both for-profit and not-for-profit organisations are involved in such practices:

when we talk about blood products and plasma products, they’re very different. When you talk about a labile blood product that’s going to be mainly used for transfusion purposes and when you talk about a plasma product which is basically a product which is a finished pharmaceutical, it’s a product that has undergone a manufacturing cycle, it’s a stable product. So it’ll have much longer shelf-life and they are products that circulate on the international level, whereas labile blood products tend to be products that have much shorter shelf-life that are used on a national scale. So that’s one of the key differences. Another difference, I would say lies with collection practices, for both blood and plasma products. Blood products, obviously they come from blood donations made by blood donors. Plasma products, most of the plasma products used on a global scale come from plasmapharesis donors. So these are people who actually donate the plasma but not the whole blood. They go to specialised plasmapharesis centres [… this] accounts for approximately 70 percent of the plasma that is used for therapeutic purposes on a global scale. And then the 30 percent of the remaining plasma comes from plasma that has been recovered from blood donation. (Int. 16, Executive of an international patient organisation)

In the UK, blood donation policy and the use of plasma products has been shaped by concerns about the potential risks of transmission of the prion disease vCJD. As we explain, plasma obtained from *male* whole blood donors may be used to produce fresh frozen plasma (FFP) for transfusion in adults but no British plasma is used to manufacture plasma products. As a consequence many plasma products and tons of plasma for use in UK plasma product manufacture are imported.^2^ It is a policy that is regarded as ‘silly’ by another respondent who saw blood and plasma donation in Europe differently:

Most European countries get their plasma from volunteer non-remunerated donors. […] And there’s no way you can get a safe plasma supply if you pay the donors and unfortunately Britain is now importing plasma in the hundreds of thousands, hundreds of tons, from plasma donors in the US, which is a disgrace. There’s no other word for it. And you are undermining the self-sufficiency of Europe because of your, I have to say, silly rules, about not using British plasma. (Int. 14, International donor organisation)

The principle of national self-sufficiency in blood supply and plasma products has been considered an important way of maximising blood safety but, as this plasma manufacturer explained, manufacturing processes have advanced and plasma products are: ‘Very safe. Very safe. In terms of viral transmission … [and] in terms of infectivity the products are very, very safe’ (Int. 27, Plasma manufacturer). He described how the US donor management programmes enhance the safety profile of sourced plasma within the global industry. With respect to the prevention of transmission of the prion implicated in vCJD, his view was that: ‘The technology for removing those is only just being developed’ and would need to be suitable for use in large-scale manufacturing processes. Therefore, governing risk in blood services and the plasma industry poses technical challenges but also raises ethical and political concerns. The commonly expressed view above (typical in Europe) is that paying donors increases the potential threats to blood safety and is unethical. Moral issues are deeply entwined with constructions of risk and safety.

While many national blood services are founded on voluntary unremunerated donation, closer analysis of the literature reveals how, within transfusion science and blood policies, categories of donor and recipient bodies construct moral hierarchies and scales of exchange and use value which have excluded MSM and migrants as donors, and segregated blood along racial lines in some countries (Bekker and Wood, 2006; Martucci, 2010; Starr, 1998; Valentine, 2005). Polonsky et al. say:

In some instances the gifting of blood offers a way for citizens to literally become a part of their nation, imagine a link between countrymen who could be ‘blood brothers’ and to feel socially included and part of the political process. (2011: 337)

Interestingly, while attending to the exclusion of migrant peoples and their citizenship status, Polonsky et al. fail to recognise the gendered notion of citizenship operating here. For example, in societies where marriage is the key source of status and security for women, those who are carriers of thalassemia are marginalised and socially excluded (Chattopadhyay, 2006). In these contexts, women’s reproductive role continues to be associated with social exclusion and marginalisation, rendering them as ‘not quite citizens’. Other studies have highlighted the gendering of blood donation. In Brazil, only a third of donors are women and donation is understood as a form of ‘bloodletting’ for those who use hormone contraceptives to suppress menstruation (Sanabria, 2009). In Pakistan, women’s blood is regarded as ‘impure’ and they are not expected to donate blood except when no men are available (Mumtaz et al., 2012). Experiences of blood donation are gendered too. Blood may be seen both as self and as alienable and rendered ‘non-self’ following manipulation and transfusion. The notion that blood becomes anonymised and ‘just a product’:

was a distinctly masculine idea among the regular blood donors. Eight out of nine male blood donors used some version of this model to understand what they were doing when they gave blood, whereas the female donors, with the exception of the nurse, were more inclined to think of their blood as forming *a relationship* with the recipient, either as a kind of loan or as a shared substance (Waldby et al., 2004: 1468, emphasis added)

Our evidence indicates that women are constructed as different kinds of donors and patient-recipients in relation to the assessment of risk within this blood economy. As we describe below, the gender identity of blood travels with it to the processing centre, and the maternal body represents a threat to blood and plasma product safety.

## Plasma – A Global Business

In the UK, blood collection is organised through four national blood services based on unpaid, anonymised, voluntary donation. Alongside this collection of whole blood there is a global plasma products industry. Plasma product manufacturers obtain plasma from both source companies, as well as ‘recovered’ plasma from whole blood collected by blood services and hospitals (Figure 1). Plasma products are exported around the world for the treatment of diverse medical conditions. Much of the plasma is collected in the US from paid individuals but there are a number of countries, such as those in the Benelux region, which operate on a not-for-profit basis, sourcing plasma from voluntary, non-remunerated donors.

Demand for plasma and plasma-derived products is on the increase, with one estimate putting global demand for plasma for fractionation (Figure 1) at 41.7 million litres by 2015 (O’Mahony and Turner, 2010: 447). Plasma supply is price sensitive and maintaining the supply of plasma donors (and blood donors) is a key priority for both national governments and the industry. In the UK, plasma is sourced from the US for use in fractionation since the exclusion of all UK donors for the production of plasma products over ten years ago. However, supplies of FFP are obtained from whole blood donations within the UK blood services.

For the most part, unlike state-sponsored blood services, which emphasise the ethics of voluntarism and altruism, the for-profit plasma products industry has not made claims to universality or social citizenship in soliciting plasma donations. Indeed, the predominantly US-based industry has traditionally relied on the socio-economically disadvantaged or marginalised to provide plasma on a regular basis in return for cash. While historically women’s plasma has been valued where they carry high titres of RhD antibodies which can be used in the manufacture of Anti-D Ig, the industry has shown a long standing preference for male plasma, targeting prisoners, inmates in mental institutions and gay men for antibodies needed to manufacture particular products, such as gamma globulin and the hepatitis B vaccine (Starr, 1998). Donor compliance and reducing ‘risky behaviour’ is at the heart of plasma procurement practices today. There are industry standards which apply to the procurement, processing and manufacturing of plasma which are intended to reduce the risk of transmissible infections and viral disease, and improve the quality and safety of the product (Farrell, 2012). Voluntary standards have been developed through the International Quality Plasma Programme and, in Europe, the sourcing of plasma must comply with the European Blood Directive.^3^ There has also been more stringent oversight at national and supranational level to ensure compliance with manufacturing standards within the scope of international pharmaceutical (medicinal products) regulation (Farrell, 2012).

What we want to draw attention to here is that plasma technologies and products are inscribed with gender, that the technologies of risk calculation are not neutral but are socially embedded. The continuing debate about the risks attributed to MSM, and their exclusion as blood (and tissue) donors since the HIV crisis, has drawn attention to the contingent and discriminatory practices associated with the risk calculus involving blood safety (Galarneau, 2010; Hoeyer, 2010). Within transfusion science, blood relations and exchanges are seen by Strong as creating ‘vital publics’ which are ‘embodied associations elicited through the generalized exchange of body – in this case blood. Participation in the vital public might therefore be seen as a unique duty associated with the biological citizen’ (Strong, 2009: 173). He argues that exclusions of MSM as donors relied on technologies which first sought to measure risk, then model it mathematically. Yet the assessment of the risk of transfusing transmitted viral infection is problematic, and socially relative, serving the political ends of sustaining the blood supply and protecting public health. As a consequence, we suggest that policies which mobilise particular understandings of the potential risks of using plasma from women must also be viewed in this light. Although women are recruited as whole blood donors, their donations have different exchange value to those of men.

## Women Donors – Different but Equal?

Despite the appearance of the universality of blood donation, from 2003 all the UK blood services introduced a policy of ‘male donor preference’, which involved women’s plasma being discarded following whole blood collection unless a shortage of male donor supply required stocks to be supplemented. The policy was based on the view that data relating to the incidence of TRALI were linked to transfusion with plasma from women (Chapman, 2011). This potentially fatal ‘syndrome’ has only been characterised and named over the past 20 years and definitions of it have been the subject of international debate and discussion. The clinical symptoms of breathlessness, hypoxia and pulmonary changes are associated with other causes of lung trauma, making definition and diagnosis difficult (Chapman et al., 2009), and pathogenesis of TRALI is poorly understood (Shaz et al., 2011). One theory put forward is that it is related to the interaction between donor HLA antibodies and leucocytes of the patient but patient factors may also be significant. There is no specific diagnostic test for TRALI.

Nevertheless, since 1996, the UK haemovigliance scheme, Serious Hazards of Transfusion (SHOT), has recorded reports of TRALI. In 2003, based on recommendations by SHOT, the National Blood Service (NBS) decided to exclude the use of female plasma for the production of FFP and suspension of buffy coat derived pooled platelets as far as possible, as part of their ‘risk reduction strategy’ (Chapman and Williamson, 2008). In 1999, SHOT introduced a set of four categories of TRALI cases indicating the level of uncertainty about the diagnosis: highly likely, probable, possible and unlikely. Each year, therefore, a high proportion of reported TRALI cases are categorised as possible or unlikely to be TRALI. A ‘consensus conference’ in Canada in 2004 described the clinical criteria of ‘TRALI’ and ‘possible TRALI’. It is the presence of antibodies in female plasma which was believed to be ‘implicated’ in the cause of TRALI. HLA antibody production is in turn associated with pregnancy in women.^4^

Despite the clinical and technical uncertainties surrounding the definition and diagnosis of TRALI, the association with HLA antibody-positive female donors led to this new policy and operational strategy in the following way:

Since new donor questions were being introduced regarding West Nile virus and severe acute respiratory syndrome, it was agreed that no additional questions would be asked regarding previous pregnancy or transfusion. As a result of the option appraisal, the NBS introduced in October 2003 a policy to use male plasma for manufacture of FFP as far as was operationally possible. This policy did not involve any additional donor questioning or loss of any donors from either whole blood or apheresis collections, since plasma is not collected by the latter method. Fractionation of UK plasma was discontinued in 1999 because of vCJD, so all plasma from non-FFP donations is discarded.

(Chapman et al., 2009: 443)

Being female was therefore defined as a risk factor, in a similar way to potential prion transmission from donors. *All* women, whether they had a history of pregnancy or not, were, as a result of this new policy, deemed unfit (or at least undesirable) for plasma donation. From this time onwards, whole blood donations were labelled ‘M’ or ‘F’ at the donor session for easy identification at the processing centre. Since 2010, 100 percent of FFP has been produced from recovered male plasma in the UK (NHS Blood and Transplant, 2012). At its largest national blood processing centre, female plasma, once it is separated from the red blood cells at the first stage of processing, is redirected to the ‘discard’ section to be sent for incineration. Risk factors are associated, not with reproductive history per se, but with reproductive *potential*, materialising female bodies as inherently ‘risky’ sources of plasma.

It has been suggested that this ‘rather crude approach’ to the exclusion of female plasma was ‘simple and low cost to implement’ (Chapman et al., 2009: 449). Moreover, because of the changes to policy relating to fractionation of UK plasma in the wake of vCJD:

‘male FFP’ could be implemented without loss of any donors and without the need to discuss the policy with the company undertaking fractionation. Donors were already aware that plasma from most donations was discarded and we have not specifically communicated the male plasma policy to them. Similarly, the decision to suspend most BC-derived PLT [buffy-coat-derived pooled platelet concentrates] pools in male plasma could be implemented relatively easily.

(Chapman et al., 2009: 449)

Although all female plasma was to be discarded at the first stage of processing following donation, it was not considered necessary by UK blood services to inform women donors that this was to take place. This appears to have been rationalised on the basis that the services did not want to adversely affect their willingness to donate blood. This presumes that some women might decide not donate blood if they knew that all their plasma was discarded and that women have no entitlement to such information.

In addition, due to exclusion policies relating to vCJD risk, FFP for patients born after 1996 in the UK has been imported since 2004 from *male* donors in the US, so these patients are not transfused with UK plasma.^5^ Products derived from plasma pools are also expected to be derived from male donors known as ‘Donor 1’. Hence staff at the processing centre we visited referred to the ‘superior’ qualities of male plasma and men who donate. In short, the change in UK policy appeared to raise no political concerns and, because it was easily implemented alongside other restrictions relating to the use of UK plasma, it received no public attention. From the point of view of UK blood services, it was easy to discard female plasma and this was seen as neither wasteful nor deserving of wider public justification or explanation.

In information provided to donors, NHS Blood and Transplant explains how plasma is used but does not make it clear either that no plasma from UK donors is used in fractionation or that its ‘male preference policy’ operates in a way where women’s plasma is seldom used at all:

Once separated from blood cells, plasma for patient use goes in one of two directions. It can be used for blood transfusion as fresh frozen plasma and other transfused plasma products. It can also be directed to a plasma fractionation plant to undergo a more complicated type of processing to separate out its many individual proteins.^6^

Such a statement lacks transparency and appears to deliberately obscure practices within the UK blood services regarding the use of plasma, and the use of female plasma in particular.

Under the cloak of changes which recognised that UK donors were implicated as at risk of transmitting vCJD, and therefore their plasma had limited exchange value, the specific gendering of policy relating to plasma processing was effectively obscured. Unlike the discrimination against MSM, this policy exclusion of women’s plasma was invisible for, though appearing to treat female donors as equal to male donors, exclusion criteria operated *after* donation, at the stage of processing blood donations. In short, it serves to perpetuate the myth of universality even though in reality only certain ‘extractions’ from women are retained for use in transfusion. Women’s bioavailability was considered jeopardised by their potential to become pregnant, which, in turn, could increase antibody production. In the circumstances, FFP and other pooled platelet suspensions are now highly gendered productions in the UK and elsewhere.

Strategies to prevent TRALI have been implemented in diverse ways in other countries, based on differing interpretations of the evidence of the link with female donor plasma. In Australia, for example, the Australian Red Cross Blood Service now has a policy to use predominantly male-only plasma for fresh plasma products. In its publicly available Factsheet, it cites the UK experience as the evidence base, while also acknowledging scientific uncertainty about the causes of TRALI (Australian Red Cross Blood Service, 2012). In the US, account is taken of both the diverse causes of TRALI (Chapman, 2011) and scientific uncertainties surrounding the syndrome. US proposals highlight the need for a ‘partial, incremental approach in making policy decisions’ and suggested ‘a plasma diversion strategy’, which diverted female plasma for fractionation and used male plasma for transfusion, but which sought to avoid alienating and confusing female donors (Eder et al., 2007). In Canada, a combination of preference for male plasma transfusion and high-volume plasma components, and screening of women who have a history of pregnancy, has been thought to improve transfusion outcomes (Lin et al., 2012).

Such measures have been designed to retain women donors while also adopting a risk reduction strategy. Yet one study suggested that cardiac patients who received female plasma transfusion had better outcomes and that excluding female plasma donors could have detrimental effects (Welsby et al., 2010). Others argued that evidence supported female donor screening and exclusion (Shaz et al., 2011). Paradoxically, while the gender identity of FFP is considered important in relation to TRALI, in a recent study of the use of FFP in England which questioned its clinical benefits, no data on the gender of patients receiving it were collected (Stanworth et al., 2011; Yang et al., 2012).

In sum, men and women are viewed as very different kinds of donors within transfusion science. Women may be regarded as potentially risky plasma donors, due to their antibody status, which is presumed in the blanket exclusion of female plasma wherever possible in the UK. Women are neither routinely screened nor tested for their antibody status. This contrasts with other western countries which use a different approach, one that redirects rather than discards female plasma, or screens women donors for their pregnancy history. Maternal bodies and maternity are represented as a potential threat to the safety of the plasma supply.

## Women Recipients of Anti-D Ig

So far we have considered the gendering of donation; the gendering of transfusion practices, however, extends to women as recipients of blood and plasma products, particularly in the obstetric context. More specifically, the implications of pregnancy for women’s need for transfusion or plasma products, as well as their status as blood or plasma donors, is a defining feature of blood relations. This is the case not only for those who become pregnant, have a miscarriage or abortion, but for all women. What is of interest here is the treatment of a relatively small group of women who are blood type RhD negative (an estimated 16% in the white UK population; NICE, 2008; Pilgrim et al., 2009), which has wider significance for how women are positioned within the blood economy.

In pregnancy, where the woman is RhD negative and the fetus is RhD positive, if fetal maternal haemorrhage occurs where fetal cells cross the placenta, it may stimulate antibody production which can cause Rh HDFN, a potentially fatal blood disorder (NHS Blood and Transplant, 2010). The production of HLA antibodies by the woman, known as sensitisation, may have particularly serious consequences for subsequent pregnancies, and therefore the administration of a plasma product, Anti-D Ig, is designed to prevent sensitisation and antibody production. Anti-D Ig can be administered prophylactically antenatally or postnatally or following any sensitising event which may have caused fetal maternal haemorrhage (this might include miscarriage, abortion, or intrauterine testing and trauma). Although Anti-D Ig has been used as a prophylactic treatment for over 40 years in many western countries (Kumpel, 2001, 2002; Kumpel and Elson, 2001), the mechanism of this passive immunisation is poorly understood and the efficacy of different treatments has not been well studied (Pilgrim et al., 2009). Nevertheless, it is considered to have been highly successful in reducing the incidence of HDFN. Paternal blood group testing may assist in identifying those women at risk of becoming sensitised. A new technology for antenatal testing involving cell free fetal DNA (cffDNA), which circulates in the maternal blood (genotyping), has the potential to identify the blood group of the fetus and therefore determine whether there is a need for prophylactic administration (Brojer et al., 2005; NICE, 2008). To date, this test has not become widely available for ‘at risk’ women. However, there are risks for women associated with the use of Anti-D Ig and its incorrect administration, and many women receive this product unnecessarily (Bolton-Maggs et al., 2013; Finning et al., 2008; Kent et al., 2014).

In Ireland, between 1977 and 1994, large numbers of women were infected with hepatitis C (HCV) as a result of receiving contaminated Anti-D Ig product manufactured by the Blood Transfusion Service Board (BTSB), which ran the national blood service at the time. The Anti-D Ig in question had been sourced from two female Irish donors who had received plasma exchange treatment. The BTSB was committed to achieving national self-sufficiency in blood and plasma products and failed to withdraw the products even after those in charge of the service became aware that various batches were likely to have been contaminated with hepatitis. Following the introduction of testing on all blood donations for HCV in the early 1990s, cases involving women blood donors who were HCV-positive emerged, leading to an investigation which revealed the extent of HCV contamination of Anti-D Ig.

A high-profile political scandal followed. Positive Action, a support group for women affected by the contamination, successfully lobbied the government for a comprehensive health care package, and for a tribunal to be established to award financial compensation for the harm they had suffered (Positive Action, 2012). The Irish government also convened a Tribunal of Inquiry, which found that there had been a clear contravention of policy guidelines in relation to both the way in which blood was collected as source material for Anti-D Ig and the protocols used in the manufacture of the product by the BTSB (Tribunal of Inquiry, 1997: 148). A programme to trace and identify all the women who received HCV-contaminated Anti-D Ig found that 1089 women were HCV antibody positive, of whom 503 are positive on polymerase chain reaction testing with viral type consistent with infection through Anti-D Ig (Irish Blood Transfusion Service, 2012). The Irish case highlighted the consequences of viral transmission through plasma products at a time when new technologies for viral testing were emerging, and the vulnerability of pregnant women receiving Anti-D Ig therapy.

## The Use of Anti-D Ig in the UK

The use of Anti-D Ig in the UK today raises complex safety issues and risks for women related to its misuse and the product itself. In 2011, 249 adverse events involving administration of the product were reported to SHOT (SHOT, 2011). Sixty women received Anti-D Ig when they should not have been given it, while in 157 cases it was omitted or given later than recommended. As one interviewee noted:

A very good friend of mine is a midwife and she regularly administers Anti-D. I asked her do you understand that this is a pooled blood product and if one of your patients asked you what are the risks would you know? She didn’t. (Int. 2, Government adviser)

More recently, administration of Anti-D Ig has become a target for training of midwives and will shortly be the subject of a national audit in the UK. Although there are no direct health benefits to women from routine administration of Anti-D Ig, and despite some criticism (Wickham, 2001), the prevention of HDFN remains a priority and justification for its use (Harkness, 2012; Harkness et al., 2008; NICE, 2008). However, current UK guidelines recommend that *all* RhD negative women should be offered prophylactic Anti-D Ig, which means that those who carry a RhD negative fetus (approximately 40,000 women per year) receive this product unnecessarily (Finning et al., 2008).

Anti-D Ig is commercially produced, has a very large market and the costs to health services of routine antenatal Anti-D prophylaxis are high (NICE, 2008). Since administration of Anti-D Ig became routine, the numbers of sensitised women whose blood may be used as source material for the product has been reduced, Anti-D Ig is currently commercially produced from predominantly *male* bodies. Male plasma donors, who are well known to the source company and have well characterised plasma, constitute a pool from which potential Anti-D donors are selected.^7^ They are paid a premium to receive repeated doses of RhD positive red blood cells to stimulate an immune response to produce antibodies which can then be harvested at the next plasma donation. Anti-D Ig is then manufactured from pooled plasma from these men for use in women. (It may also be used infrequently in cases where RhD negative men are wrongly transfused with RhD positive blood.)

This procedure is unusual in a number of respects for it is the only active intervention in the body of a male plasma donor to stimulate antibody production. Second, it attracts a market premium and is tied in to a scheme of incentives for men who donate plasma which is unavailable to paid women donors, unless they are ‘older’ and post-menopausal (though few of these are used).^8^ The higher price paid for this plasma reflects potential risks to the male donor but also the higher value placed on their bodily products. Women who produce these antibodies naturally (as a result of pregnancy) are excluded and women of reproductive age are regarded at high risk of sensitisation which could adversely impact on future pregnancies. The technology to produce monoclonal (synthetic) Anti-D Ig has been developed, but is not currently commercially viable (Kumpel, 2001, 2002; Interview 27).

What is revealed here is how transactions between men and women are technologically mediated. These technologies are embedded within a gendered blood economy where male bodies are sourced for plasma and plasma proteins and the production of commercially available Anti-D Ig, shaped by an immuno-politics which merits closer analysis.

## Maternity and the Immune System

Transfusion science draws on a set of rationalities about the immune system which have emerged within a specifically gendered historical context (Haraway, 1995). The biomedical body was conventionally seen to represent the nation-state and invading organisms as foreigners. More recently, scientific and cultural understandings see the immune system as a complex networked system (Martin, 1994). Within this framework, maternal bodies, in particular, are often represented as paradoxical and explanations about why the genetically distinct fetus is not rejected by the mother have provoked considerable debate and stimulated a long history of investigation. In pregnancy the fetal and maternal circulations have increasingly been understood as closely interconnected. Maternal fetal microchimerism refers to the mixing of cells from the maternal and fetal body (Kelly, 2012; Martin, 2010) and placental ‘border crossings’ which may provoke an immunological response. Kelly’s (2012) account of the emergence of gestational cell transfer science and understandings of maternal–fetal microchimerism reveals the extent of scientific uncertainty about the significance and meaning of these exchanges. While previously thought to be pathological, fetal cells entering the maternal circulation have more recently been considered normal.

Biology has intrinsically been a branch of political discourse which constructs and constitutes political subjects (Kelly, 2012: 5). Immunology theory supported and reinforced a set of political values about normal and pathological states, subjectivity and identity. The fetus was thought of as a hostile ‘invader’ or ‘intruder’ and the placenta as a barrier between two separate selves (Martin, 1994). The dominant view was that the immune system ‘defends the self’ and the pure body is the normal body (Kelly, 2012). With recognition that cells routinely cross the placental interface, a relational model of the immune system begins to emerge, as a complex, dynamic network (Haraway, 1995). Instead of a barrier, the placenta may be viewed as a permeable interface where exchanges between mother and fetus occur in both directions – ‘bidirectional cell trafficking’ (Martin, 2010). The discovery of the intermingling of maternal and fetal circulations and blood type incompatibility as a cause of HDFN in the 1960s was important in developing Anti-D Ig therapy. The implications of a revised model of the immune system – as relational and networked – are much wider. First, the notion that *all* bodies are chimeric undermines notions of the modern political subject as a distinct individual (Kelly, 2012); second, it implies a different way of thinking about antibodies, and their significance for transfusion practices starts to seem far more complex than a blanket exclusion of women as plasma donors in the UK suggests. Yet, in the context of transfusion science, it is presumed that the immune status of (all) women is compromised because of their potential exposure to a fetal body, thereby affecting their suitability to become plasma donors. In contrast, the commercial production of Anti-D Ig by injecting male RhD negative plasma donors’ with RhD positive cells to produce antibodies, is an accepted and valued practice.

## Conclusion

Our reading of the UK ‘male preference policy’ for plasma is that it is discriminatory. It constructs all women as risky plasma donors regardless of their age, reproductive history or antibody status. Implemented with no public consultation or discussion, there is a continuing lack of public information or explanation of this policy. This in turn raises questions about the ethics of donation – the information given to women about becoming blood donors. While retaining women donors in the donor pool may be an important policy objective, the different ways in which their donations are processed is information which should be made available. The myths of universality, citizenship and solidarity should be publicly elaborated upon to explain and justify the ways in which women are treated as different kinds of donors from men. As we have shown, women are unable to donate blood as frequently as men; all women’s plasma is likely to be discarded in the UK; and elsewhere its use is limited to processing for fractionation.

We have also shown that Anti-D Ig is produced mainly from male bodies for use in pregnant and parturient women. Women of reproductive age are usually excluded from becoming donors for the product or having access to additional payments for such donations. Moreover, many women receive Anti-D Ig when it is unnecessary (Kent et al., 2014). In short, the institutional arrangements for plasma collection and processing favour ‘Donor 1’ men. Practices around procurement and distribution render gender differences both invisible and visible. Patient information describes Anti-D Ig as produced from ‘specially selected donors’ (NHS Blood and Transplant, 2010) and women are often unaware that they may not need Anti-D Ig if they are carrying an RhD negative fetus. Plasma products are not simply homogeneous, neutral, universal technologies – rather gender is inscribed upon them. Gender difference is produced within the industry and blood services.

Technologies of risk assessment are also not neutral. The calculation of risk associated with FFP plasma transfusion rests on limited understandings of the causes or characterisation of TRALI and an evidence base which includes a wide-ranging categorisation of ‘highly likely, probably, possible, unlikely’ cases. Transfusion science, as a discourse of truth and power, is tied to technological change and emergent forms of knowledge and expertise. In creating categories of donors, donor deferral policies, screening, testing and matching policies, it ‘governs life’ through the exercise of biopower. Women’s position is tied to their reproductive work and their reproductive potential within an increasingly globalised and commercial blood economy (Busby et al., 2013), in circumstances where the policy rationale for such positioning remains unclear and lacks transparency.

## Notes

1. ESRC Project RES-062-23-2751. Ethical approval for the study was given by an NHS REC.

2. The sale of Plasma Resources UK in July 2013 to Bain Capital has been especially controversial (see: https://www.gov.uk/government/news/minister-welcomes-bain-capital-buying-majority-stake-in-pruk; http://www.opendemocracy.net/ournhs/lucy-reynolds/selling-our-safety-to-highest-bidder-privatisation-of-plasma-resources-uk).

3. See: http://www.pptaglobal.org; Directive 2002/98/EC setting standards of quality and safety for the collection, testing, processing, storage and distribution of human blood and blood components and amending Directive 2001/83/EC OJ L 33.

4. Multiparous women are more likely to have high levels of antibodies (Chapman, 2011).

5. Joint UKBTS/NIBSC Professional Advisory Committee (2007) Position statement: Creutzfeldt-Jakob Disease. SHOT Report (2003: 63).

6. See: NHS Blood and Transplant ‘How blood is used’, http://www.blood.co.uk/about-blood/how-blood-is-used/plasma (accessed August 2013).

7. Men who have been previously transfused are excluded.

8. See advertising for RhD negative plasma donors – by Cangene Plasma, Biotest which recruits women and men; women must be surgically sterile or post-menopausal.
